# Evaluation of the Predictive Character of the Expiratory Inferior Vena Cava/Abdominal Aorta (eIVC/Ao) Index for Minimum Blood Pressure Following Spinal Anesthesia With 0.5% Hyperbaric Bupivacaine

**DOI:** 10.7759/cureus.67310

**Published:** 2024-08-20

**Authors:** Justin Merle-Béral

**Affiliations:** 1 Anaesthesia, Saint-Pierre University Hospital, Brussels, BEL

**Keywords:** inferior vena cava aorta index, bupivacaïne hyperbaric, anesthesia spinal, low bp, drop in bp, fasting

## Abstract

Spinal anesthesia has many side effects, one of them being a drop in blood pressure (BP). Identifying predictive factors for this drop is a clear matter of concern. In this regard, the expiratory inferior vena cava/abdominal aorta (eIVC/Ao) index has already been spotted as such for doses of 0.5% hyperbaric bupivacaine greater than 12mg. Departing from the demonstrated correlation between this index and hypotension post-spinal anesthesia, our study aimed to (1) evaluate whether an eIVC/Ao index greater than 0.7, thus defining non-hypovolemic patients, can also predict minimal BP for doses inferior to 12mg and (2) identify other predictive factors for minimal BP post-spinal anesthesia. Lastly, we verified whether preoperative fasting induces hypovolemia.

This single-center prospective observational pilot study included 20 patients. The baseline measurements of BP, eIVC/Ao index, and fasting time were recorded at time T0'. Then spinal anesthesia was administered with 0.5% hyperbaric bupivacaine in doses inferior to 12 mg. The patients’ systolic blood pressure (SBP), diastolic blood pressure (DBP), mean arterial pressure (MAP), and metameric levels were each recorded at times T5', T10', T15', and T20'.

The results indicated that baseline DBP was predictive of low DBP and minimum MAP, which reflect myocardial perfusion and systemic pressures, respectively. Therefore, it should trigger prophylaxis (spinal-lateralized, continuous, or lower dose) in patients with a low DBP baseline. Additionally, baseline SBP was predictive of minimum SBP, an independent risk factor for post-anesthetic hypotension if its baseline is less than 120 mmHg. Although female gender was linked to minimum SBP, other confounding factors (size, dose administered, and type of surgery related to gender) must also be considered. Moreover, a correlation was established between height and MAP in parturients. Hypotension was not recorded at local anesthetic (LA) doses between 8 and 12 mg and the doses administered were sufficient to achieve the metameric levels required for surgery (ether tests). Since 8 mg of 0.5% hyperbaric bupivacaine achieved the same level as 12 mg, lower doses of LA might prevent a significant drop in BP and its deleterious effects.

Therefore, in the current cohort, the eIVC/Ao index was not predictive of minimum BP during spinal anesthesia with doses less than 12 mg of 0.5% hyperbaric bupivacaine. However, predictive factors for minimum BP included gender and baseline SBP (for minimum SBP), height and baseline DBP (for minimum MAP), and baseline DBP (for minimum DBP). Lastly, preoperative fasting did not cause hypovolemia.

## Introduction

Spinal anesthesia is associated with a significant drop in blood pressure (BP) to the point of hypotension (defined as systolic blood pressure (SBP) < 90 mmHg and/or diastolic blood pressure (DBP) < 60 mmHg [1]). Hypotension occurs in 15.3%-33% of cases [2] (particularly in pregnant women) and can interfere with tissue perfusion, leading to significant morbidity [3]. 

The index of the diameter of the inferior vena cava in exhalation compared to that of the abdominal aorta in systole (the expiratory IVC/Ao index) is a rapid, simple, non-invasive, and reliable method of determining the volume status of patients [4-6]. The pilot study by Salama et al. comprising 100 patients undergoing spinal anesthesia showed that the eIVC/Ao index is a predictive factor for the occurrence of post-spinal anesthesia arterial hypotension [7]. However, the doses of 0.5% hyperbaric bupivacaine were greater than 12 mg in the study. 

The present study aimed to evaluate whether an eIVC/Ao index > 0.7 ± 0.09 [4] was predictive of minimal post-spinal anesthesia BP for < 12 mg of 0.5% hyperbaric bupivacaine in non-hypovolemic patients. In addition, we also determined whether there were other factors predictive of minimal post-spinal anesthesia BP and whether the duration of preoperative fasting-induced hypovolemia.

## Materials and methods

Herein, we conducted a single-center, prospective, observational pilot study at University Hospital Saint-Pierre, Brussels (Belgium) including 20 patients. The study was approved by the ethics committee (BE0762023231202).

The demographic characteristics recorded were sex, age, height, weight, body mass index (BMI), and American Society of Anesthesiologists (ASA) score.

The inclusion criteria were as follows: age between 18 and 65 years, ASA score I-II, and scheduled lower limb, urological, gynecological, or digestive surgery that could be performed under spinal anesthesia with 0.5% hyperbaric bupivacaine.

The exclusion criteria included patient refusals, contraindication to spinal anesthesia, lateralized spinal anesthesia, pediatric and geriatric populations, emergency surgery, patients with extremes of stature, i.e. too short (<160 cm) or too tall (>190 cm), use of sedatives, intrathecal adjuvant, preoperative volume replenishment, pregnant women, patients with poor echogenicity, those treated with beta-blockers and/or angiotensin-converting enzyme (ACE) inhibitors, and those assessed as hypovolemic using the according to the eIVC/Ao index or already suffering from arterial hypotension prior to spinal anesthesia.

Preoperative recording and evaluation

**Figure 1:**
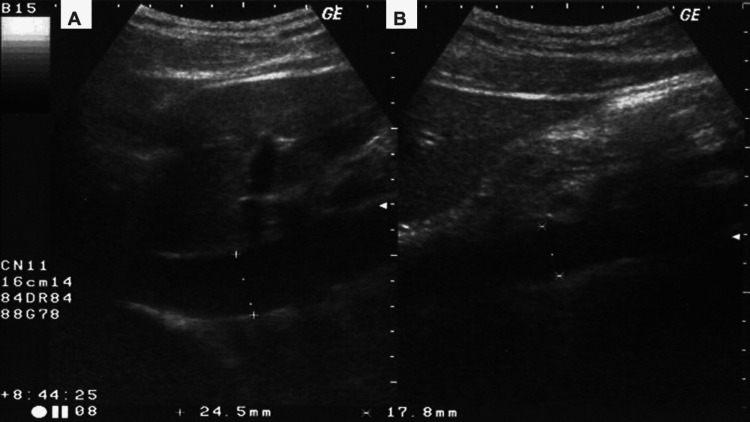
Ultrasound measurement point location for eIVC and aorta A: Expiration IVC; B: Systole aorta eIVC: Expiration Inferior Vena Cava

The duration of fasting was recorded when the patient arrived in the operating room. At T0', the anesthetist recorded the baseline BP (SBP, DBP, and mean arterial pressure (MAP)). Then, the patient's eIVC/Ao index was measured in the semi-seated position by ultrasound. In M mode (curvilinear low-frequency probe CA2-8AD, Siemens HS40 machine) in the subxiphoid window: measurement of the diameter of the inferior vena cava (eIVC) in the long axis 2 cm from the right atrial outlet. The anteroposterior systolic diameter of the aorta was estimated to be between 5 and 10 mm above the celiac trunk. Subsequently, the eIVC/Ao index was calculated as the ratio of the maximum diameter of the IVC during exhalation to the maximum diameter of the aorta during systole [6,7]. The image was recorded during three consecutive respiratory cycles in the inspiratory and expiratory phases by the same anesthetist (for the 20 cases) with a level 2 ultrasound experience and with a resident cardiologist reviewing and validating the images (Figure 1). 

After ultrasound identification of the lumbar spine in the seating position, a 27-G Whitacre needle was inserted subarachnoidally at L3-L4. Then, a dose of < 12 mg of 0.5% hyperbaric bupivacaine was injected and adjusted according to the metameric level required for surgery. Each of the SBP, DBP, and MAP values was recorded at time points T5', 10', 15' and 20', and so were the metameric levels, by ether tests. Finally, arterial hypotension, if ever occurring, was planned to be treated using vasoactive agents and/or fluid challenge as appropriate.

Statistical analysis

Pearson's correlation analysis was applied to compare the continuous data. The relationship between a continuous variable and a discrete variable was assessed using Student's *t-test* when the underlying assumptions were met: 1) homogeneity of variances was evaluated using the Bartlett test and 2) normality of residuals was assessed using the Kolmogorov-Smirnov test. The results of the Student's *t-test* were presented as the means and standard deviations per group. When the hypotheses underlying the *t-test* were not fulfilled, a Wilcoxon rank test was applied to the data, and the results were presented as medians and interquartile ranges. Finally, the data of several independent variables were analyzed using multiple linear regression models to predict the minimum BP level during the perioperative period. These data were entered into the multivariate regression analysis predicting minimum BP. For each of them, no multicollinearity was established (variance inflation factor (VIF) ≤ 5). In the case of multicollinearity, one of the two causative factors was removed from the regression model.

Next, we used a backward variable selection approach, sequentially removing the non-significant variables.

R software version 4.2.0 (R Core Team, 2021) was used to produce the statistical results [8].

## Results

A total of 24 patients were recruited in the present study. Of these, three had poor echogenicity or structure detection was not feasible, and one patient withdrew consent. Consequently, 20 patients were included in the study. Among them, three (15%) had undergone orthopedic surgery, five (25%) had gynecological surgery, nine (45%) had urological surgery, and three (15%) patients had undergone proctological surgery. The cohort comprised 11 (55%) men and nine (45%) women, and their average age was 39.4 ± 12.35 years, average height 172.8 ± 7.52 cm, average weight 76.35 ± 9.84 kg, and average BMI 25.34 ± 2.80 kg/m^2^. Moreover, 11 (55%) patients were ASA I vs. 9 (45%) ASA II.

The average duration of preoperative fasting was 13.9 ± 3.22 h for solids and 11.9 ± 4.53h for liquids. The average eIVC/Ao index was 1.07 ± 0.21. 

No hypotensive episodes were observed in the perioperative period. The minimum recorded post-spinal anesthesia SBP, the minimum MAP, and the minimum DBP were 108, 78, and 60 mmHg, respectively (Tables 1, 2).

**Table 1:** Descriptive statistics ASA:  American Society of Anesthesiologists

Descriptive statistics	Number (%)
Female	9 (45%)
Male	11 (55%)
ASA Score I	11 (55%)
ASA Score II	9 (45%)
Urological Surgery	9 (45%)
Gynecological Surgery	5 (25%)
Proctological Surgery	3 (15%)
Orthopedic Surgery	3 (15%)

**Table 2:** Recorded measurements for the Wilcoxon rank test BMI: Body Mass Index; SBP: Systolic Blood Pressure; DBP: Diastolic Blood Pressure; MAP: Mean Arterial Pressure; IVC/Ao: Inferior Vena Cava/Aorta

Variable	Min.	1st Qu.	Median	Mean	3rd Qu.	Max	SD
Age (years)	23.00	30.50	35.50	39.40	47.75	64.00	12.35
Weight (kg)	56.00	69.50	79.50	76.35	83.25	90.00	9.84
Height (cm)	160.0	167.0	173.0	172.8	178.5	183.0	7.52
BMI (kg/m^2^)	19.60	24.50	25.30	25.34	27.10	29.70	2.80
Ratio IVC/Ao	0.75	0.91	1.07	1.07	1.19	1.58	0.21
SBP Baseline (mmHg)	108.0	127.5	132.0	134.6	143.8	168.0	15.86
DBP Baseline (mmHg)	60.00	68.00	74.00	73.65	78.25	99.00	9.06
MAP Baseline (mmHg)	78.00	89.75	95.00	96.33	105.25	114.00	10.57
Solid Fasting (h)	8.00	11.88	12.75	13.85	16.00	21.00	3.22
Liquid Fasting (h)	4.00	9.75	11.75	11.85	13.00	23.00	4.53

Independently of the BP studied (systolic, diastolic, and mean) at the cuff, the eIVC/Ao index was not predictive of the minimum BP in the study population *(*if* p > 0.05)* (Tables 3-11).

**Table 3:** Pearson correlation between the minimum SBP variable and other continuous variables ASA:  American Society  of Anesthesiologists; BMI: Body Mass Index; SBP: Systolic Blood Pressure; DBP: Diastolic Blood Pressure; MAP: Mean Arterial Pressure; IVC/Ao: Inferior Vena Cava/Aorta *: p-val<0.05; **: p-val<0.01 (p-value predictive if < 0.05)

Variable	Minimum SBP
Age	0.22
Weight	0.21
Height	0.45*
BMI	-0.15
ASA	0.29
SBP Baseline	0.60**
DBP Baseline	0.29
MAP Baseline	0.38
Ratio IVC:Ao	0.07
Bupivacaïne (Dose)	0.04

**Table 4:** Comparison of minimum SBP, function of gender, and ASA score using Student's t-test ASA:  American Society of Anesthesiologists; SBP: Systolic Blood Pressure p-value predictive if < 0.05

Variable	Variable =0	Variable =1	p-value
Sex (0=F, 1=M)	113.11 ± 10.68	126.18 ± 11.06	0.01564
ASA (0=I, 1=II)	117.09 ± 8.36	124.22 ± 15.94	0.214

**Table 5:** Results of multivariable linear regression SBP: Systolic Blood Pressure Pr(>|t|) value predictive < 0.05

Variable	Estimate	Std. Error	t value	Pr(>|t|)
Intercept	56.0875	20.0951	2.791	0.01206
SBP	0.4772	0.1484	3.216	0.00479

**Table 6:** Pearson correlation between the minimum DBP variable and other continuous variables ASA: American Society of Anesthesiologists; BMI: Body Mass Index; SBP: Systolic Blood Pressure; DBP: Diastolic Blood Pressure; MAP: Mean Arterial Pressure; IVC/Ao: Inferior Vena Cava/Aorta *: p-val<0.01 (p-value predictive if < 0.05)

Variable	Minimum DBP
Age	0.23
Weight	0.01
Height	0.28
BMI	-0.27
ASA	0.31
SBP Baseline	0.43
DBP Baseline	0.63*
MAP Baseline	0.56*
Ratio IVC:Ao	0.00
Bupivacaine (Dose)	0.08

**Table 7:** Comparison of minimum DBP, function of gender and ASA score, using Student's t-test ASA:  American Society of Anesthesiologists; DBP: Diastolic Blood Pressure p-value predictive if < 0.05

Variable	Variable =0	Variable =1	p-value
Sex (0=F, 1=M)	63.56 ± 9.51	68.27 ± 8.38	0.2538
ASA (0=I, 1=II)	63.73 ± 8.13	69.11 ± 9.57	0.1901

**Table 8:** Results of multivariable linear regression DBP: Diastolic Blood Pressure pr (>| t |) predictive if < 0.05

Variable	Estimate	Std. Error	t value	Pr(>|t|)
Intercept	20.3214	13.5139	1.504	0.14999
DBP Baseline	0.6222	0.1822	3.416	0.00308

**Table 9:** Pearson correlation between the minimum MAP variable and other continuous variables ASA: American Society of Anesthesiologists; BMI: Body Mass Index; SBP: Systolic Blood Pressure; DBP: Diastolic Blood Pressure; MAP: Mean Arterial Pressure; IVC/Ao: Inferior Vena Cava/Aorta *: p-val<0.05 (p-value predictive if < 0.05)

Variable	Minimum MAP
Age	0.16
Weight	0.24
Height	0.56*
BMI	-0.21
ASA Score	0.26
SBP Baseline	0.54*
DBP Baseline	0.46*
MAP Baseline	0.46*
Ratio IVC:Ao	0.12
Bupivacaine (dose)	0.04

**Table 10:** Comparison of minimum MAP, function of gender, and ASA score using Student's t-test ASA:  American Society of Anesthesiologists; MAP: Mean Arterial Pressure p-value predictive if < 0.05

Variable	Variable =0	Variable =1	p-value
Sex (0=F, 1=M)	83.44 ± 9.76	89.32 ± 8.79	0.1741
ASA (0=I, 1=II)	84.50 ± 8.89	89.33 ± 10.00	0.2676

**Table 11:** Results of multivariable linear regression DBP: Diastolic Blood Pressure pr (>| t |) predictive if < 0.05

Variable	Estimate	Std. Error	t value	Pr(>|t|)
Intercept	-37.5776	41.8559	-0.898	0.3818
Height	0.5030	0.2331	2.158	0.0456
DBP Baseline	0.5070	0.1935	2.620	0.0179

However, two variables, height and baseline SBP, were predictive of minimum SBP. These variables were included in a multivariate regression analysis. Although the VIF was 8 for baseline SBP, baseline SBP did not detect multicollinearity between the variables (VIF close to 1) in the final model, which explained 37.91% of the variance in minimum SBP. The only variable predicting minimum SBP in a multivariate regression model was baseline SBP (*p *= 0.0151). Additionally, once controlled for baseline SBP, height did not have a significant role in predicting minimum SBP (*p* = 0.1374) and was removed from the model (Table 2). On the other hand, a correlation was established between minimum SBP and female gender (*p *= 0.01564).

Furthermore, two variables predicted minimum DBP: baseline DBP and baseline MAP. These were included in a multivariate regression analysis, and no concern for multicollinearity was detected. Then, the variable not significantly associated with the dependent variable was removed: baseline MAP (*p* = 0.484). The final model contained baseline DBP, which explained 35.95% of the variance in the minimum DBP (Tables 6-8).

The independent variables significantly associated with the baseline MAP were entered in multivariate regression analysis. A significant VIF was detected for baseline SBP, which we removed from the analysis. We also removed the variable non-significantly associated with the dependent variable: baseline SBP (*p* = 0.2555). The final model contained baseline height and baseline DBP, which explained 35.2% of the variance in minimum MAP (Tables 9-11).

## Discussion

In the present study, we did not find a correlation between the eIVC/Ao index and minimum BP during spinal anesthesia with 0.5% bupivacaine at doses < 12 mg. The pilot study by Salama et al. predicted the occurrence of arterial hypotension using the eIVC/Ao index during spinal anesthesia [7]. However, this study differs from our approach in several aspects: (1) Our study included only patients who were not hypovolemic according to the eIVC/Ao index; (2) A low dose of 0.5% hyperbaric bupivacaine injected (< 12 mg vs. 12-15 mg); (3) The average age of the patients was lower (39.4 years *vs.* 49.5 years). 

Previous studies have identified the factors associated with a drop in BP following spinal anesthesia: the dose of the local anesthetic (LA) injected, the height of the T5' block, and age of ≥ 40 years [3,9,10]. In our opinion, these methodological and demographic differences between our study and that of Salama et al. may explain the absence of a significant drop in BP in our cohort. 

Another study comprising 172 patients demonstrated that the mean eIVC/Ao index in normovolemic patients was 1.2 ± 0.12, hypovolemic 0.7 ± 0.09, and hypovolemic 1.6 ± 0.05 [4]. These results were further confirmed with an eIVC/Ao index of 1.2 ± 2 for SD = 0.17, defining a normovolemic state [6]. These findings align with the results as the mean value of the eIVC/Ao index in our cohort (1.07 ± 0.21) is compatible with a state of non-hypovolemia, which may also be termed a state of "physiological dehydration" [6]. Another recent study by Rahman et al. suggested a cutoff of the eIVC/Ao index at 1.14 ± 2 with SD 0.18 to detect the early phase of hypovolemic shock [5]. These relative discrepancies suggested that the standardization of eIVC/Ao index values concerning different volemic statuses needs an in-depth investigation.

The lack of correlation between the eIVC/Ao index and minimum BP in our cohort could be explained by the small sample size or a minimal drop in the BP.

The average age of our cohort was 39.4 ± 12.35 years, representing a relatively young population. Thus, it is not surprising that age was not a predictive factor for minimal BP after spinal anesthesia in our population. Elderly patients (defined by the World Health Organization (WHO) as individuals > 60 years old; clinical studies use a cutoff of 65 years) are at greater risk of a drop in BP following spinal anesthesia due to "closure" of the holes of conjugation linked to bone deformations [11-14]. This phenomenon might hinder the diffusion of the LA outside the medullary sheath, causing significant diffusion of the anesthetic block and other cardiovascular performance factors [9,10,13].

Similarly, obesity (defined by a BMI > 30 kg/m^2^ according to the WHO [15]) is a risk factor for post-spinal anesthetic pressure drop. Magnetic resonance imaging showed that obese people have up to 10 ml less cerebrospinal fluid than those with a normal BMI, which is a potential source of excessive spinal block for the same dose of LA, therefore causing a drop in BP [3,13]. The average BMI in our cohort was 25.34 ± 2.8 kg/m²; thus, our patients were below the risk profile.

In addition, some studies have shown that a high ASA score (> II) is a determining factor in arterial hypotension during general or spinal anesthesia; the older the patient, the greater the risk [16]. All our patients were considered to be at low risk of anesthesia (55% ASA I and 45% ASA II); therefore, our results were in line with the literature.

On the other hand, we also determined other factors predictive of minimal BP during spinal anesthesia with 0.5% bupivacaine at doses < 12 mg. Baseline DBP appeared to be a factor underlying DBP and minimal perioperative MAP. Since DBP and MAP are crucial in myocardial and systemic perfusion, respectively, patients with lower baseline DBP may be identified as requiring special monitoring during surgery [17]. 

Baseline SBP was predictive of minimal SBP post-spinal anesthesia with 0.5% bupivacaine. Hence, the anesthetic was termed an independent risk factor for the development of post-spinal arterial hypotension if the baseline SBP is < 120 mmHg [3]. 

A statistical relationship was established between minimum SBP and female gender (p = 0.01564). Also, confounding factors, such as height, dose administered, and type of surgery, could explain this result.

Finally, height also seemed to be a factor predicting minimum MAP; this relationship has been elaborated mainly in the literature on parturients [18]. 

In agreement with Muller et al. [19], we found that the recorded duration of fasting was not associated with preoperative hypovolemia. Instead, our patients had an eIVC/Ao index > 0.75, corresponding to a state of non-hypovolemia, despite fasting duration exceeding the current recommendations [20].

Furthermore, our study found no evidence of arterial hypotension (absolute minimum SBP: 108 mmHg and absolute minimum DBP: 60 mmHg) for doses of hyperbaric 0.5% bupivacaine ranging from 8 mg to 12 mg. Also, we did not observe any failure of spinal anesthesia regardless of the type of surgery considered, which was confirmed by the ether test of the metameric levels at time points T5', T10', T15', and T20'. These findings suggested that we could consider doses of 0.5% hyperbaric bupivacaine in our daily practice to obtain a sufficient metameric level in certain surgeries. Although the same metameric level could be achieved for different doses of 0.5% hyperbaric bupivacaine (8 mg or 12 mg), the duration of the sensory-motor block is altered (39 min vs. 55 min) [14]. A lower dose of 0.5% hyperbaric bupivacaine might prevent arterial hypotension and its potentially deleterious effects. Thus, the baricity of the LA and the dose are two of the main determinants of LA distribution [13,14].

Methodology limitation

The primary limitation of this study is the small sample size, which results in insufficient statistical power. Thus, future studies with larger patient cohorts are recommended to confirm or refute our findings.

## Conclusions

The eIVC/Ao index did not show any correlation with minimum BP during spinal anesthesia with hyperbaric 0.5% bupivacaine at doses less than 12 mg in non-hypovolemic patients. However, predictive factors for minimum blood pressure, under the set circumstances, were gender and baseline SBP (for minimum SBP), height and baseline DBP (for minimum MAP), and baseline DBP (for minimum DBP). Lastly, the duration of preoperative fasting in our cohort did not result in hypovolemia, according to the eIVC/Ao index.
